# Effects of enteral nutrition in stroke: an updated review

**DOI:** 10.3389/fnut.2025.1510111

**Published:** 2025-03-31

**Authors:** Dailiang Jiang, Lei Nie, Xuying Xiang, Xiaoqing Guo, Mengting Qin, Shengnan Wang, Jiaojiao Chen, Yuhang Feng, Ming Huang, Ling Mao

**Affiliations:** ^1^Department of Neurology, Union Hospital, Tongji Medical College, Huazhong University of Science and Technology, Wuhan, China; ^2^Department of Neurology, Hubei Provincial Hospital of Integrated Chinese and Western Medicine, Hubei University of Chinese Medicine, Wuhan, China

**Keywords:** stroke, malnutrition, dysphagia, enteral nutrition, prognosis, stroke associated pneumonia

## Abstract

Stroke is a leading cause of death and functional decline that places a significant burden on healthcare systems. Malnutrition is a critical clinical concern that complicates the condition of stroke patients and contributes to unfavorable outcomes. Dysphagia is the primary cause of malnutrition in the acute stage after stroke. Enteral nutrition (EN) has been employed to manage the nutritional status of stroke patients to prevent and treat malnutrition. Early EN (EEN) has been shown to reduce mortality and the prevalence of malnutrition while enhancing functional outcomes. In the majority of cases requiring nutritional support, nasogastric tube (NGT) placement is prioritized. However, under specific circumstances, direct enteral tube (DET) feeding that includes percutaneous endoscopic gastrostomy (PEG) and percutaneous endoscopic jejunostomy (PEJ), offers distinct advantages, particularly for PEG. Compared to intermittent EN, continuous EN demonstrates better tolerance. An EN protocol providing sufficient nutrient supply and energy support can alleviate neurological deficits and reduce the severity of motor dysfunction in stroke patients, thereby improving their prognosis. Energy-rich formulations of EN and EEN may be associated with a lower incidence of stroke-associated pneumonia (SAP). However, the use of EN may lead to an increased incidence of digestive complications, and hyperglycemia. In this study, we reviewed the indications, opportunities, and management methods for EN application, along with the nutrient composition of nutritional protocols for stroke patients.

## 1 Introduction

Stroke is often defined as a rapidly developing disease with characteristic symptoms of local (or global) disturbance of cerebral function, lasting more than 24 h or leading to death, with no other significant cause except vascular origin (1). As the aging population increases, the prevalence of ischemic and hemorrhagic stroke has nearly doubled over the past several decades (2). Stroke remains one of the leading causes of death worldwide. Additionally, the absolute number of disability-adjusted life years (DALYs) associated with stroke has risen significantly, particularly in developing countries, likely due to the growing number of stroke survivors (2, 3). This significant increase in chronic disability among stroke survivors, coupled with the prevalence of stroke in an aging population, has led to a rapid escalation in the burden on healthcare systems (3).

Malnutrition can occur in post-stroke patients in the acute stage, which may cause impaired motor ability and cognitive function and malrehabilitation of stroke patients (4, 5). The prevalence of moderate-to-severe malnutrition among acute ischemic stroke patients ranges from 2 to 6% (6–8), and it can even result in aspiration pneumonia (9). Dysphagia is a major contributing factor to malnutrition in stroke patients (10, 11). It is associated with a worse prognosis in stroke patients than those without dysphagia (12). In clinical practice, enteral nutrition (EN) is often employed to manage critically ill stroke patients with dysphagia or high malnutrition risk to improve prognosis (4).

EN includes nasogastric tube (NGT) and direct enteral tube (DET) [percutaneous endoscopic gastrostomy (PEG) and percutaneous endoscopic jejunostomy (PEJ)]. NGT is the most commonly used method of EN and is often recommended for critically ill stroke patients with malnutrition to improve their nutritional status. DET includes PEG and PEJ, in which tubes are directly inserted into the stomach or jejunum through a surgical procedure. Compared with stroke patients who do not receive EN, those who are fed via EN experience reduced mortality, shorter hospital stays, improved swallowing function, and greater potential for addressing malnutrition (13–17). In clinical practice, healthcare providers must often decide whether to initiate EN therapy for stroke patients with dysphagia or malnutrition. However, there is still a lack of comprehensive and robust integration of existing evidence, which is crucial for making informed nutritional treatment decisions for stroke patients. Additionally, several important considerations must be addressed in the application of EN, such as identification of the appropriate patient populations, timing, management methods, and early identification and treatment of EN-associated complications. Our aim is to review the various factors related to the use and management of EN in stroke patients and to provide support for clinical practice.

## 2 Methods

**Figure 1:**
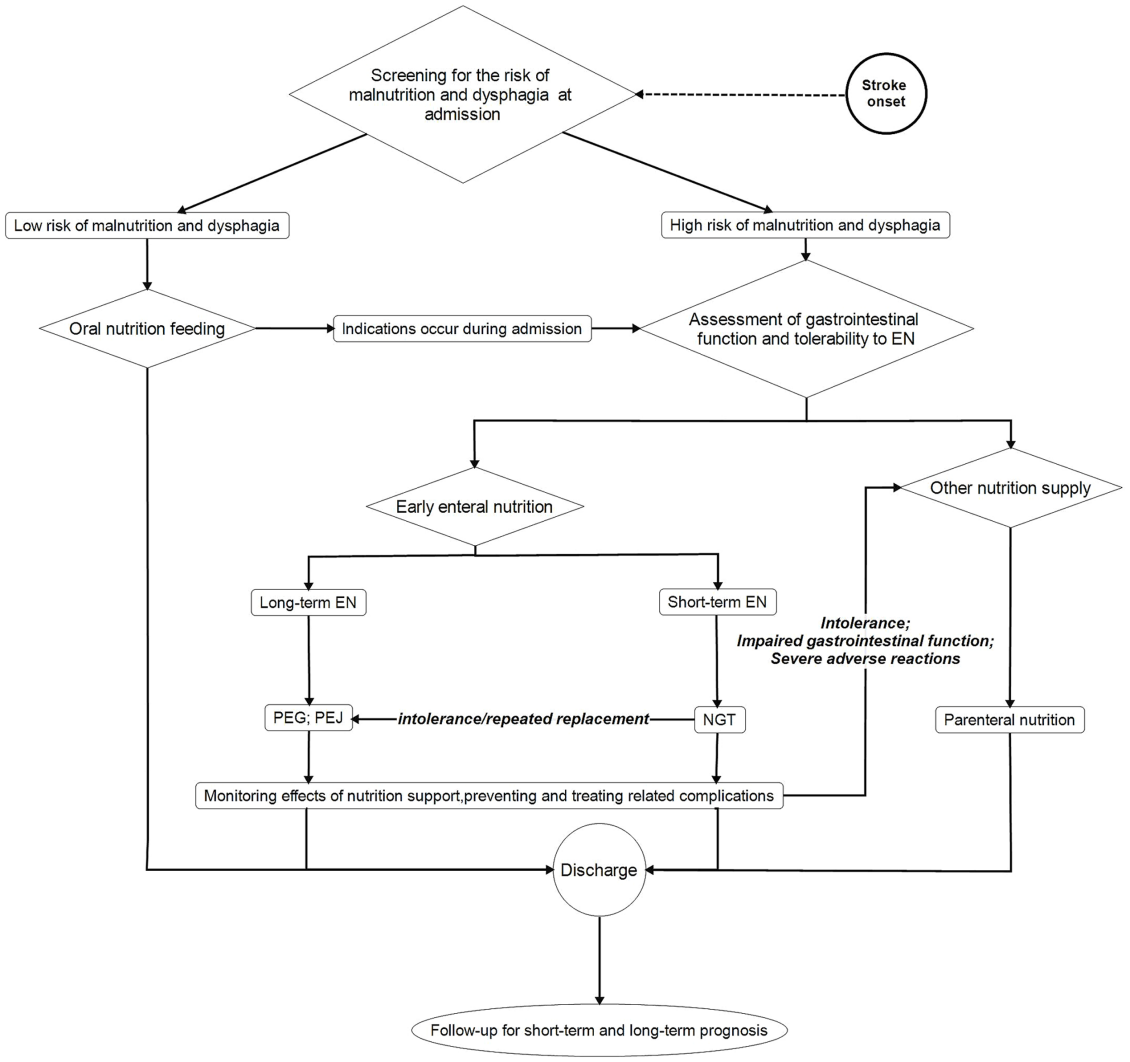
EN management procedure in stroke patients EN, enteral nutrition; PEG, percutaneous endoscopic gastrostomy; PEJ, percutaneous endoscopic jejunostomy; NGT, nasogastric tube.

We conducted a comprehensive search of the literature in PubMed and identified the following keywords: stroke, EN, prognosis, malnutrition, dysphagia, stroke-related pneumonia, diarrhea, constipation, nutrients, protein, energy, hyperglycemia, complications, and guidelines. Ultimately, we selected 87 articles for this review. Additionally, we used XMind to create Figure 1.

## 3 Which stroke patients require EN?

### 3.1 Malnutrition

Malnutrition refers to a state characterized by a deficiency, excess, or imbalance of energy, protein, and other nutrients (18). Dysphagia is a significant contributor to malnutrition in stroke patients, as it adversely affects their ability to eat and ultimately leads to a substantial reduction in nutrient intake. Severe stroke conditions can also play a critical role in malnutrition because they increase nutrient consumption and impair digestive system function. In clinical practice, several screening tools can be used to identify patients at risk for early malnutrition. These include the Nutritional Risk Screening (NRS 2002), the Malnutrition Universal Screening Tool (MUST), the Short-Form Mini Nutritional Assessment (MNA-SF), and the Short Nutritional Assessment Questionnaire (SNAQ) (19–22). Malnutrition can exacerbate adverse functional outcomes and poor health status in stroke patients, leading to increased mortality, a higher incidence of severe disability, prolonged hospital stays, greater reliance on long-term care, and numerous complications, such as pneumonia, various infections, and gastrointestinal bleeding (23–28).

### 3.2 Dysphagia

Dysphagia is a common complication in stroke patients, with a prevalence ranging from 20 to 80%. It is associated with a high incidence of malnutrition, increased mortality, a high incidence rate of aspiration pneumonia, a high proportion of discharge to acute care facilities rather than home, and a diminished quality of life (12, 29, 30). Approximately 30% of patients with dysphagia require EN management because of malnutrition caused by severe dysphagia (12, 29, 30). Dysphagia is one of the primary contributors to malnutrition in stroke patients (10, 11). Prompt initiation of EN at the onset of dysphagia is an effective strategy to prevent malnutrition. Methods to screen for dysphagia include the water swallow test (WST) and the swallowing provocation test (SPT) (31, 32). The standard definition for dysphagia screening in the WST is that coughing or hoarseness occurs within 1 min after swallowing 3 oz. of water continuously (31). Another common and convenient screening method, the SPT, involves observing the latency time of swallowing after injecting distilled water into the pharynx (32). Diagnostic tools for dysphagia include the videofluoroscopic swallowing study (VFSS) and the flexible endoscopic evaluation of swallowing (FEES) (33–35). VFSS is an accurate diagnostic method that uses barium mixed with various liquids and foods, enabling real-time visualization and recording via X-ray (33). FEES involves the direct insertion of an endoscope into the pharynx to assess the swallowing function (34, 35). Additionally, the predictive swallowing score (PRESS) has been developed as a prognostic model for dysphagia following stroke and may help predict the recovery of swallowing function (36).

### 3.3 Other stroke patients requiring EN

Other scenarios that may necessitate EN include patients with reduced levels of consciousness and those requiring mechanical ventilation because these patients cannot eat properly. However, these indications have not been adequately substantiated by sufficient evidence from clinical studies (37). Research suggests that patients with high National Institutes of Health Stroke Scale (NIHSS) scores, large lesions, or lesions located near the middle cerebral artery may be candidates for EN management (38, 39). Complete digestive function is essential for nutrient absorption; therefore, assessing digestive function is a critical component in determining the feasibility of EN. If digestive function is compromised, total parenteral nutrition (TPN) should be considered as the primary option because it provides complete nutrition through intravenous injection, independent of the digestive system.

## 4 Opportunities, insertion pathways, and continuity in EN management

### 4.1 EEN can improve stroke patient outcomes

EEN refers to the initiation of EN within 72 h of identifying the risk of dysphagia or malnutrition. For the majority of stroke patients who need to be fed by EN without contraindications, EEN should be initiated as early as possible (4). In the FOOD trial, the early placement of an enteral tube was found to be associated with a decreased mortality rate and fewer poor outcomes, although the difference was not obvious (14). Meanwhile, EEN could lower the incidence rates of various infections, especially stroke-associated pneumonia (SAP), and significantly improve neurological function compared with late EN (LEN) (13, 15). The length of stay (LOS) in the hospital and the LOS in the intensive care unit (ICU) in the EEN population are shorter than those in the LEN population in three retrospective observational studies (15, 16, 40). Another retrospective observational study reported that the proportion of discharge to home, rather than other long-term medical institutions, was higher in patients fed with EEN (41), which may indicate that patients who were fed with EN were more likely to gain better daily life ability and functional recovery. The reason why EEN improved the prognosis and outcomes of stroke patients may be attributed to the fact that it can provide adequate nutrients or energy in the early stage of acute stroke, which may be beneficial for post-stroke repair of neurological system function. Altogether, EEN may be advantageous in enhancing the prognosis of stroke patients. Therefore, EEN is deemed to be an appropriate choice for stroke patients who require urgent nutritional support. These EN methods are summarized in Table 1.

**Table 1:** Opportunity, route, continuity, and composition for the utilization of EN in stroke patients (4, 13–15, 43, 47, 55, 60, 63, 86, 87).

	Methods	Reasons and explanations
Opportunity	Initiation of EN <3 days after screening for swallowing function, along with the risk of malnutrition (4)	EEN can improve functional outcomes and decrease mortality and the incidence rate of complications (13–15)
Route	NGT (with nasal loops)	NGT can improve the prognosis of stroke patients with relative safety Nasal loops help to prevent dislodgment and extubation (43)
	DET (especially PEG)	DET is the better choice when NGT cannot supply enough nutrition, intolerance to NGT exists, or long-term nutrition support is necessary (4)
Continuity	Continuous EN	Continuous EN decreases the incidence rate of intolerance to EN (47)
Composition	Sufficient energy supply	Sufficient energy supply can improve prognosis, swallowing function, ADL, and functional outcomes in stroke patients (86)
	Sufficient protein supply and whey protein	Sufficient protein supply can decrease mortality and improve functional outcomes (86, 87)
		Whey protein has been shown to have antioxidant and anti-inflammatory functions (55)
	EN protocol with probiotics, prebiotics, or dietary fiber	Probiotics, prebiotics, and dietary fiber are beneficial in preventing digestive complications and promoting the prognosis of stroke patients (60, 63)

EN, enteral nutrition; EEN, early enteral nutrition; NGT, nasogastric tube; DET, direct enteral tube; PEG, percutaneous endoscopic gastrostomy; ADL, activities of daily living.

### 4.2 Application of NGT and direct enteral tube (DET)

#### 4.2.1 NGT

NGT has long been the most widely used form of EN in inpatients due to its convenience, safety, and effectiveness. It can be used to improve the nutritional status of stroke patients in the acute stage or to prevent the worsening of malnutrition (42). In randomized controlled trials, NGT has shown its effectiveness in decreasing mortality and improving prognosis in stroke patients (13, 14). However, its disadvantages are notable, such as frequent replacement, dislodgement, self-extubation, and tube clogging. A randomized controlled trial demonstrated that a greater likelihood of gastrointestinal hemorrhage was associated with stroke patients fed with NGT compared to the group fed with PEG (14). A nasal loop has been proven to be safe and tolerated in acute stroke patients and could be utilized in short-term EN to ensure the fixation of NGT, consequently reducing the likelihood of repeated dislodgement of the NGT in a prospective cohort trial (43). It is important to note the occurrence of nasal mechanical complications, such as nosebleeds, although the severity is not pronounced (44).

#### 4.2.2 DET

Compared with NGT, DET offers an advantage in long-term nutritional support. The likelihood of PEG intervention failure (such as feeding interruption, blocking or leakage of the tube, and no adherence to treatment) is lower than that of NGT (45). Moreover, a randomized controlled study found that the efficacy of PEG in the rehabilitation of dysphagia and improvement of nutritional status was superior to that of NGT (17). However, a multicenter randomized controlled clinical trial found a borderline significant increase in the absolute risk of death or poor outcome in stroke patients fed through PEG compared with the group fed through NGT (14). A retrospective study found that the LOS in the stroke and dysphagia patient groups fed through PEG was still longer than in those fed through NGT (46). Evidently, it is caused by a more severe stroke in patients fed through DET. There is little evidence regarding the cost-effectiveness of both NGT and PEG methods in stroke patients. Additional studies are necessary to obtain sufficient evidence for further discussion about the cost of EN for stroke patients.

Generally, when EN is required during the acute stage after a stroke and the nutritional needs can be met through NGT delivery, it is preferred over DET. However, if this approach fails to achieve the nutritional target, if long-term nutritional support is essential for severe stroke, or if NGT intolerance persists after multiple attempts, PEG is a more favorable option compared with other EN methods.

#### 4.2.3 Continuous EN can decrease the incidence of digestive symptoms

Intolerance to EN is frequently defined as the appearance of certain digestive symptoms or complications, such as diarrhea and constipation. In a randomized controlled clinical trial, a comparison between patients receiving continuous EN and those receiving intermittent EN showed no significant difference in the incidence of vomiting, abdominal distension, constipation, gastric retention, and gastrointestinal bleeding (47). However, the overall intolerance rate of EN and the incidence of diarrhea were found to be lower in the continuous EN population compared to the intermittent EN population (47). These findings suggest that continuous EN may be an effective strategy for improving the tolerance of stroke patients to EN and reducing the incidence of diarrhea in stroke patients. Since critically ill stroke patients account for a high proportion of the EN-fed group, their compromised gastrointestinal function may not be able to handle large volumes of EN solution in a short period during a single intermittent feeding activity. In the majority of clinical scenarios, continuous EN is recommended for stroke patients because it enhances efficacy and reduces the incidence of EN-associated digestive complications (37). More effective methods are required to improve EN tolerance in stroke patients receiving EN.

## 5 Effects of energy, protein supply, dietary fiber, prebiotics, and probiotics in EN protocols

### 5.1 Sufficient energy supply is necessary for stroke patients

In a multicenter, prospective, randomized trial, participants were divided into three groups: EN with full energy supply (70–100% of energy requirements), prokinetic agents (modified full EN group), and hypocaloric EN (40–60% of energy requirements) (48). The final results of this study indicated that the hypocaloric EN protocol did not improve post-stroke outcomes or reduce the incidence of complications compared with the other two groups (48). The risk of death was relatively higher in the hypocaloric EN group than in the modified full EN group (48). A retrospective study found that increasing energy intake at admission may be independently associated with improved activities of daily living (ADL) after stroke (49). Another retrospective study suggested that higher energy intake may be linked to better ADL outcomes and a lower incidence of complications, such as pneumonia and urinary tract infection, following a stroke (50). However, since these studies did not only target stroke patients fed with EN, the above results might need to be further researched in stroke patients fed with EN (49, 50). The amount of energy supplied by EN can be accurately measured using indirect calorimetry, which is considered the best method for determining energy requirements (51). Other simplified approaches to assessing energy supply include calculating it using predictive equations or estimating it based on a reference of 20–25 kcal/kg body weight per day or 25–30 kcal/kg body weight per day; however, these methods are not as accurate as indirect calorimetry (51, 52).

### 5.2 Sufficient protein supply contributes to stroke rehabilitation

For protein nutrition, the supply should be estimated based on the patient’s clinical condition. In a retrospective observational study of stroke patients in the neurocritical care unit, researchers found that both short- and long-term mortality exhibited a downward trend as the protein supply increased until it reached 1.74 g/kg body weight per day, which can be considered the potentially appropriate point of protein supply (53). In an investigator-initiated, double-blind, multicenter, parallel-group, randomized controlled trial, researchers found that patients in the ICU fed by EN with higher enteral protein provision (~1.87 g/kg body weight per day) were associated with worse prognosis and a higher incidence of symptoms of gastrointestinal intolerance than those fed with EN with standard enteral protein provision (~1.19 g/kg body weight per day) (54). Current guidelines recommend 1.3 g/kg body weight per day as an appropriate supply in critical illness (51). In a randomized controlled clinical trial comparing various protein types, patients who received whey protein exhibited increased levels of glutathione peroxidase (GPx) and reduced serum interleukin-6 (IL-6) levels. These findings suggest that whey protein enhances antioxidant capacity and mitigates inflammatory responses (55). The reduction in inflammation and oxidative stress, along with the increase in antioxidant activity, may help prevent further inflammatory damage to the nervous system after a stroke. This mechanism can be attributed to the high leucine content in whey protein, a branched-chain amino acid (BCAA) that modulates inflammation by maintaining plasma glutamine concentrations and regulating inflammatory cytokines such as tumor necrosis factor and interleukin-1 and -4 (56).

Another retrospective observational study demonstrated that an EN protocol enriched with leucine could improve the nutritional status of stroke patients (57). Based on current evidence, EN protocols containing whey protein or other proteins rich in BCAAs may offer advantages for stroke patients.

### 5.3 Dietary fiber, prebiotics, and probiotics can decrease the incidence of digestive symptoms and promote recovery from neurological deficits

Dietary fiber is generally recognized for its role in aiding digestion and nutrient absorption, which can help alleviate digestive symptoms. Prebiotics are beneficial, non-digestible food ingredients that improve host health by promoting the growth and activity of various bacteria in the colon (58). Dietary fiber encompasses all types of prebiotics and certain non-prebiotic nutrients (59). For the majority of patients who receive EN, a non-fiber protocol can increase the incidence rate of constipation and diarrhea, so an EN protocol containing enough soluble fiber or prebiotics may be associated with a lower incidence of these digestive complications (60). Several studies have shown that prebiotics and other dietary fiber can promote the growth and reproduction of short-chain fatty acids (SCFAs)-producing bacteria in the gastrointestinal microbiome. SCFAs have been proven to promote nervous system function and support cognitive rehabilitation (61, 62). Regulation of the production of SCFAs may be a potential mechanism by which prebiotics are beneficial to stroke recovery. Given these benefits, dietary fiber is recommended for use in daily nutritional protocols for patients requiring EN management (52).

Probiotics, which refer to various microbial food supplements that are beneficial to health, are another potential nutrient in EN protocols that may improve outcomes for stroke patients (58). The addition of probiotics to EN protocols has been associated with a lower incidence of gastrointestinal complications, reduced rates of dysbiosis, fewer infections, and shorter hospital stays (63). Commonly studied probiotics, such as Bifidobacterium, belong to the category of SCFA-producing bacteria and may enhance stroke prognosis (64). While the use of probiotics in other diseases has been linked to increased levels of SCFAs (65), which may explain the beneficial effects of probiotics for stroke recovery, research on the application of probiotics in nutritional support following a stroke remains limited. EN protocols with probiotics remain scarce, and further studies are needed to evaluate the efficacy of probiotics in post-stroke nutritional treatment regimens.

## 6 The effect of EN on post-stroke complications

### 6.1 SAP

The incidence rate of SAP in stroke patients ranges from 11 to 44% (66, 67). The majority of cases of SAP are diagnosed in the early stage after stroke (66, 68). A retrospective cohort trial reported that stroke patients affected by SAP suffer from high mortality, longer LOS, poor neurological system function, and poor prognosis (67). Weakened defenses and post-stroke immunosuppression may also be an important cause for SAP. A retrospective observational clinical study indicated that EEN can decrease the incidence rate of SAP (15). In another retrospective study, the EN group with a more adequate energy supply had a lower incidence of pneumonia than the lower energy supply EN group (50). Overall, based on the current evidence, energy-rich formulations of EN and EEN may be associated with a lower incidence of pneumonia.

### 6.2 Digestive complications

#### 6.2.1 Diarrhea and constipation

Overall, EN increases the incidence of diarrhea and constipation compared to oral nutrition (69). Diarrhea is defined as the evacuation of three or more watery stools within 24 h. A higher incidence of diarrhea was observed in stroke patients fed via EN for >7 days compared to those fed for <7 days, suggesting that a temporal cutoff point may exist at 7 days (69). This finding indicates that controlling the duration of EN may help prevent diarrhea. Another bowel motility dysfunction is constipation, which is defined as the evacuation of less than once in 3 days. Constipation can negatively impact the quality of life, limit social activities, and lead to adverse outcomes, such as disability, poor neurological function, and increased mortality (60, 70). A meta-analysis found that the incidence of constipation in stroke patients is 48% (70). Patients on non-fiber EN protocols are more likely to experience diarrhea or constipation than those receiving fiber supplementation (60). A gastrointestinal microbiome disorder may be present in stroke patients, which may be an important cause of diarrhea and constipation, and EN protocols containing prebiotics may reduce these complications by promoting the growth of commensal bacteria in the intestinal microbiota.

#### 6.2.2 Gastrointestinal hemorrhage

Another challenge associated with EN is gastrointestinal hemorrhage (71). EN is believed to increase the incidence of gastrointestinal bleeding, which is more common in patients receiving EEN compared to those receiving LEN (14). The NGT group exhibited an association with a higher incidence of gastrointestinal hemorrhage than the group fed by PEG (14). The use of NGT and DET can cause mechanical damage to the gastrointestinal mucosa, potentially increasing the risk of gastrointestinal bleeding. Additionally, anticoagulants and antiplatelet agents are frequently used in acute stroke patients to prevent embolism, which can also contribute to gastrointestinal hemorrhage (72, 73). The protocols commonly used to treat gastrointestinal bleeding include two types of medications: acid-suppression drugs, such as proton-pump inhibitors (PPIs), and hemostatic agents that affect the clotting process or enhance coagulation to stop the bleeding (74). Furthermore, the addition of fiber may effectively reduce the incidence of gastrointestinal hemorrhage (63).

### 6.3 Correlation between EN-associated hyperglycemia and poor prognosis

Among all hospitalized patients, the incidence rate of hyperglycemia is >30% (75). The incidence of hyperglycemia is especially high in patients fed with EN, up to 40%, which may lead to an elevated risk of mortality in stroke patients (76, 77). Patients affected by hyperglycemia during EN exhibited a low rehabilitation rate of swallowing function (77). However, a clinical study showed that many patients affected by hyperglycemia had suffered from this problem before EN (78). Insulin has been considered a key medication for controlling hyperglycemia in stroke patients. Insulin is used to control blood sugar levels within a relatively safe range. However, a clinical study revealed that irrespective of the route of administration, insulin cannot effectively maintain blood sugar levels in a low range (79). Furthermore, excessive insulin administration can elevate the risk of hypoglycemia (generally defined as a blood glucose level of <3.9 mmol/L in the group receiving EN) (80), potentially increasing the risk of malnutrition and hindering rehabilitation. Using a computerized monitoring system to adjust insulin dosage may be an effective strategy to control blood sugar levels (81). Another important method of managing EN-associated hyperglycemia is the diabetes-specific formula. In a randomized, prospective, controlled trial, the postprandial glucose parameters, such as capillary glucose concentration, from 8 to 16 h after EN consumption, incremental area under the curve, peak value, and mean glucose concentration were significantly lower in the group fed with a diabetes-specific EN formula than in the group fed with a standard formula (82). The diabetes-specific EN formula is suitable for EN protocols for stroke patients with hyperglycemia.

### 6.4 Other associated complications

Enteral tubes frequently encounter blockages that diminish nutrient and energy supply, escalating the risk of unfavorable outcomes. A pump system provides a solution to this challenge by automatically flushing the enteral tube, thereby demonstrating its capacity to reduce the incidence of mechanical tube blockages (83). Although the use of EN may be associated with these complications, it is recommended that stroke patients require nutritional support (51, 84). Implementing appropriate EN protocols and management strategies can effectively prevent complications rather than avoiding the initiation of EN altogether.

## 7 Effective EN management procedure

An overview of effective EN management in stroke patients is shown in Figure 1. When stroke patients are admitted, assessment for dysphagia, malnutrition, and decreased level of consciousness is essential to identify patients who require EN early and to avoid the incidence of various complications (4). After screening for the above indications, if an EN protocol is necessary, EN should be immediately initiated (85), while if patients do not require nutritional support, they can be fed with family-managed nutrition. Even for stroke patients initially considered low-risk, if they shift to a high-risk category or exhibit signs of dysphagia or malnutrition, EN remains a viable option. If possible, EN protocols should contain as much energy, protein, prebiotics, and other dietary fiber as possible. Doctors should assess how long patients need EN. In cases requiring short-term EN, nasogastric tube (NGT) placement is preferred. For patients requiring long-term EN, PEG, and PEJ are preferable for sustained nutritional support (4). If intolerance or frequent NGT replacement occurs, priority should be given to percutaneous endoscopic gastrostomy (PEG) or percutaneous endoscopic jejunostomy (PEJ) (4). In cases where stroke patients requiring nutritional support cannot tolerate EN due to factors such as compromised gastrointestinal function or severe adverse reactions, alternative feeding methods should be used. After EN initiation, frequent monitoring is an effective method to prevent complications. Prompt medical intervention for complications, such as stroke-associated pneumonia (SAP), digestive complications, and hyperglycemia, should be initiated immediately upon their occurrence. Long-term follow-up helps identify the prognosis of stroke patients, thereby assisting clinicians in making appropriate clinical decisions.

## 8 Conclusion

In conclusion, the appropriate use and management of EN can enhance the prognosis of post-stroke patients. Stroke patients may be affected by malnutrition and dysphagia and require EN. In these patients, EEN exerts a better impact on prognosis than LEN. NGT is a common method of EN. When NGT intervention has failed or long-term nutritional support therapy is needed, DET can be used for EN support therapy. Continuous EN may be better tolerated by stroke patients than intermittent EN. Overall, EN protocols containing an adequate supply of energy and protein may aid in the recovery of neurological function after stroke. A hypoglycemic formula can be used for stroke patients with hyperglycemia to help maintain steady blood glucose levels. EN protocols containing prebiotics may reduce the incidence of diarrhea and constipation. Energy-rich formulations of EN and EEN may be associated with a lower incidence of pneumonia. In clinical practice, healthcare providers must select the optimal EN protocol based on each patient’s specific condition and closely monitor for EN-associated complications. Further research is essential to gather additional evidence and to establish the best practices for EN management in stroke patients.
